# Comparison of Flowmetric Plethysmography and Forced Oscillatory Mechanics to Measure Airway Hyperresponsiveness in Horses

**DOI:** 10.3389/fvets.2020.511023

**Published:** 2021-02-22

**Authors:** Claire E. Dixon, Daniela Bedenice, Melissa R. Mazan

**Affiliations:** Department of Clinical Sciences, Cummings School of Veterinary Medicine at Tufts University, North Grafton, MA, United States

**Keywords:** bronco-alveolar lavage, lung function testing, airway hyper responsiveness, horses, inflammatory airway disease, equine asthma

## Abstract

Airway hyperresponsiveness (AHR) is linked to airway inflammation and is considered a key manifestation of mild/moderate equine asthma (EA). The study purpose was to determine whether two modalities of non-invasive lung function testing (FOM—forced oscillatory mechanics vs. FP—flowmetric plethysmography) establish the same clinical diagnosis of AHR in horses, using histamine bronchoprovocation. Nineteen horses (3–25 years, 335–650 kg) with clinical signs suggestive of mild/moderate equine asthma were enrolled. FOM and FP testing was performed in each horse on two consecutive days, using a randomized cross-over design. AHR was defined by the histamine dose needed to double FOM baseline resistance, or to achieve a 35% increase in FP delta flow. Bronchoalveolar lavage fluid (BALF) was subsequently collected and stained with modified Wright's and toluidine blue stains. Binary statistical tests (related samples *T*-test, Mann-Whitney *U*, Chi-square analyses) were performed to compare study groups, with *P* < 0.05 considered significant. Abnormal BALF cytology confirmed EA in 14/19 (73.7%) horses. Both FOM and FP revealed AHR in 7/14 (50%) of these EA horses. An additional 4/19 (21.1%) horses showed AHR based on FP but not FOM, including two horses with normal BALF cytology. A diagnosis of AHR was more often associated with FP than FOM (*P* = 0.013), although the prevalence of AHR was significantly higher in EA vs. non-EA horses, regardless of testing methodology. The phase angle between thoracic and abdominal components of breathing did not differ between test groups. In conclusion, FP diagnosed AHR more frequently than did FOM, including horses with no other diagnostic evidence of EA. Without further evaluation, these two testing modalities of AHR cannot be used interchangeably.

## Introduction

Airway hyperresponsiveness (AHR) is linked to airway inflammation and is considered a key manifestation of mild/moderate equine asthma or inflammatory airway disease (hereafter termed EA) in horses with chronic cough or exercise intolerance (1) as well as in humans with the similar disease, asthma. Despite this well-established link, the exact mechanism is still not fully understood (2). The diagnosis of equine asthma (1) requires, in addition to appropriate history, a documentation of lower airway inflammation based on bronchoalveolar lavage fluid (BALF) cytology or abnormal pulmonary function testing (PFT) demonstrating AHR. Due to lack of availability of the latter, BALF cytology is more commonly used in clinical practice (1).

AHR is defined as an exaggerated bronchoconstrictive response to specific or non-specific inhaled stimuli that is present only in patients with asthma and not in normal individuals (3), and, in the horse, is diagnosed most commonly by identifying increased sensitivity and responsiveness to the bronchoconstrictor agent histamine. Various methods of pulmonary function testing to document this bronchoconstrictive response are available in horses; however, invasive methods (such as esophageal balloon-pneumotachography) are more difficult to use in client-owned animals. Two non-invasive testing methods that can be used to assess AHR in client-owned horses are forced oscillatory mechanics (FOM) and flowmetric plethysmography (FP). FOM superimposes oscillations of compressed room air on spontaneous breaths in awake horses to measure total respiratory system resistance (R_rs_) which reflects the degree of airway obstruction. FP combines external sensors placed on the body surface (inductance bands) measuring thoracic and abdominal excursions (plethysmographic flow), and a pneumotachograph measuring airflow at the nares. Airway obstruction is determined by subtracting peak plethysmographic flow from peak nasal flow during expiration—a variable defined as delta flow (Δ_flow_). This variable is an established measure of airway obstruction and, similar to respiratory system resistance, increases with bronchoconstriction (4).

When compared to the conventional method (esophageal balloon-pneumotachography), a 100% increase in R_rs_ (PC_100_R_rs_; PC—provocative concentration) for FOM and a 35% increase in Δ_flow_ for FP were previously found to correlate with a 65% decrease in dynamic compliance (PC_65_C_dyn_) (5, 6). Changes in dynamic compliance (C_dyn_) (esophageal balloon-pneumotachography) correlate closely with inhaled histamine concentrations (7). As such, AHR is conventionally assessed by the histamine concentration needed to reach PC_65_C_dyn_, which lies outside the range of variability and occurs on the steepest portion of the histamine dose-response curve (8). Normal horses are not considered to reach PC_65_C_dyn_ until concentrations of >8 mg/mL histamine have been inhaled (7). Likewise, normal horses experience a 100% increase in R_rs_ (PC_100_R_rs_ measured by FOM) (5) or a 35% increase in Δ_flow_ (PC_35_Δ_flow_ measured by FP) (4) at a nebulized histamine dose >8 mg/mL (FOM) and >6 mg/mL (FP) (9), respectively. In contrast, horses with EA may respond at histamine concentrations below these cut points, consistent with AHR (a key component of EA). However, whilst several studies have used these definitions of AHR (10, 11), other authors have used different test end-points, including PC_75_R_rs_ measured by FOM and PC_50_Δ_flow_ measured by FP (12).

Although both FOM and FP have been separately validated against conventional esophageal balloon-pneumotachography, FOM and FP have not been directly compared, compounding difficulties in evaluating studies using either methodology to assess AHR. While each system has its advantages and limitations, FP has the benefit for clinical practice of being non-invasive and easily portable. For example, the repeatability of FP over time was determined in a large population of university horses in the field setting (4), which would have been impractical with either FOM or the conventional methodology. Our current study therefore aims to validate FP as a method of portable lung function testing by demonstrating that comparable results may be achieved using either of the available non-invasive testing modalities (FOM vs. FP) to measure AHR. We hypothesized that when compared directly, FOM and FP would identify AHR at comparable doses of nebulized histamine in horses with clinical signs of equine asthma, thus validating the use of either method to diagnose AHR in clinical cases.

## Materials and Methods

Twenty-two adult horses (200–650 kg, 2–25 years old) with clinical signs suggestive of EA (poor performance, exercise intolerance, cough, or nasal discharge) were recruited from a referral population for enrolment in a prospective crossover assessment of AHR. Horses with clinical signs of infectious disease (as determined by anamnesis, pyrexia, or physical examination) or uncontrolled severe equine asthma [recurrent airway obstruction (RAO) with signs of respiratory abnormalities at rest] were excluded from the analysis. The experimental protocol was approved by the Institutional Animal Care and Use Committee (IACUC) of the Cummings School of Veterinary Medicine at Tufts University and completed following written client consent. Detailed anamnesis was obtained using a standardized questionnaire, followed by a complete physical and respiratory examination. Further diagnostic procedures were performed if indicated (thoracic radiographs, lameness evaluation, blood sampling, echocardiography) to rule out other causes of poor performance or respiratory disease.

Horses were randomly assigned to undergo histamine bronchoprovocation using both FOM and FP with a 20–30 h wash-out period between tests. The horses were not accustomed to the mask or bands for either testing method prior to the study. All horses remained hospitalized throughout the study period and were fed according to their individual management protocols. After completion of lung function testing (day 2), a bronchoalveolar lavage (BAL) was performed, as specified below.

### Forced Oscillatory Mechanics (FOM)

Mono-sinusoidal, multifrequency (1–3 Hz) FOM was used to measure total respiratory system resistance (R_rs_) as previously described (13, 14). In brief, horses were sedated with 0.5 mg/kg xylazine^1^ intravenously (IV), administered 6 mL phenylephrine^2^ to each nasal passage *via* a small catheter, and fitted with a latex-sealed low dead space facemask, *via* which sinusoidal flow (generated using compressed air released through a proportional pneumatic valve) was superimposed over the horses' spontaneous breathing. A pneumotachograph^3^ was used to measure flow at the mask opening, and the difference between mask and atmospheric pressures recorded using a pressure transducer^4^. For each frequency, coherence was calculated, with values >0.9 being accepted for analysis. After baseline measurements were obtained, changes in R_rs_ were used to monitor effects of histamine aerosol challenge (see below) (14). During all measurements, the head rested on a stand in a neutral position.

### Respiratory Inductive Plethysmography (FP)

FP was performed using a commercial system^5^ as previously described (6). Briefly, each horse was sedated with xylazine^1^ (0.5 mg/kg IV), administered 6 mL phenylephrine^2^ to each nasal passage *via* a small catheter, and fitted with a low-dead space face mask, pneumotachograph, and thoracic (11th intercostal space) and abdominal (directly behind the 18th rib) inductance bands. Airflow and thoracic/abdominal volume changes were simultaneously measured during spontaneous breathing, and mathematically converted to plethysmographic flow signals. Airway obstruction (delta flow, Δ_flow_), thoraco-abdominal synchronicity (phase angle or Φ theta) and ventilatory parameters were recorded. Delta flow is the value recorded by the proprietary software by subtracting the flow signal generated by the thoracic and abdominal volume change at the body surface (measured by inductance bands) from the pneumotachograph flow at peak expiration (4, 6).

Thoraco-abdominal synchronicity, or phase angle (theta), is calculated by plotting the abdominal and thoracic inductance bands on x-y coordinates and using Lissajous figures as described previously (15, 16). Normal synchronous breathing has a phase angle close to 0°, which increases with increasing airway obstruction and resulting asynchrony, eventually reaching 180° which indicates complete paradoxical motion of the rib cage and abdomen. Respiratory rate was calculated breath by breath and recorded by the software, as were tidal volume, peak inspiratory and expiratory flows, minute ventilation, and inspiratory and expiratory times. During all measurements, the head rested on a stand in a neutral position.

### Histamine Bronchoprovocation (for Both FOM and FP)

Histamine bronchoprovocation was performed as previously described (14). Initial baseline lung function parameters were recorded *via* FOM or FP (R_rs_ and Δ_flow_, respectively). Nebulization of 2 mL of 0.9% saline (negative control or saline baseline) over 2 min using a low dead-space face mask with a portable air compressor^6^ and nebulizer^7^ was performed and repeated for subsequent increasing concentrations of histamine diphosphate^8^ in saline solution (2, 4, 8, and 16 mg/mL). Lung function measurements were repeated following each nebulized dose until either a 100% increase in R_rs_ from saline baseline (FOM PC_100_R_rs_) or >50% increase in Δ_flow_ (FP PC_35_Δ_flow_) was achieved, or the horse displayed a clinical reaction to histamine administration such as a notably increased respiratory rate or effort, or repeated coughing.

Following FOM testing, a dose-response curve was generated for R_rs_ at 1 Hz frequency to determine the histamine dose required to reach a doubling in saline baseline resistance (PC_100_R_rs_) for each horse. Similarly, FP data were further evaluated using a breath-by-breath analysis, to discard signals that were obviously erroneous (e.g., first breath of recording was abnormally short, tidal volume non-physiologically small or large). The delta flow for each histamine concentration was calculated by the software by discarding the two highest values and then averaging the next five highest values. Respiratory rate and theta changes were generated manually in the same way. This enabled measurement of the peak response to each histamine dose, whilst still excluding outliers. The PC_35_Δ_flow_ (35% increase in delta flow from saline) was then recalculated from a manually-derived dose-response curve. Dose-response curves for respiratory rate and theta were similarly generated.

Using the dose-response curves generated, AHR was defined when the PC_100_R_rs_ was reached at ≤8 mg/mL for FOM, or the PC_35_Δ_flow_ was reached at ≤6 mg/mL for FP.

### Bronchoalveolar Lavage and Evaluation of Bronchoalveolar Lavage Fluid

After all PFTs were completed, horses were sedated with detomidine^9^ (0.01 mg/kg) and butorphanol^10^ (0.01 mg/kg) IV and bronchoalveolar lavage performed using a commercial cuffed BAL tube^11^ or endoscope^12^ as previously described (17). A total of 120 mL of 0.3% lidocaine^13^ was used to anesthetize the upper respiratory tract and bronchi during passage of the tube or endoscope. Once wedged in a bronchus, two aliquots of 250 mL saline^14^ were instilled and then drawn back by suction at 10-cm H_2_O pressure^15^. The two samples were pooled and 10 mL added to a red top glass tube which was refrigerated and submitted within 4 h of collection for cytology. Slides were prepared after cytocentrifugation and stained with Wright-Giemsa and toluidine blue stains. Cells (*n* = 500) were classified by one of the authors (MRM) as the percentage of total cells that were macrophages, lymphocytes, neutrophils, eosinophils, and mast cells (400x magnification). Horses were classified as having an abnormal BALF if there were >5% neutrophils, >2% mast cells, or >1% eosinophils, and these horses were diagnosed with EA (1).

### Statistical Analysis

Sample size calculations estimated that 16 horses would provide sufficient statistical power (alpha = 0.05 and power >85%) to reject the null hypothesis of test equivalence (FP vs. FOM) if the alternative is true. All continuous data were presented descriptively as mean ± SD or median ± range, and compared between test groups using related samples *T*-test or Mann-Whitney *U*-test, based on the normality of data distribution (Kolmogorov-Smirnov analysis). Chi squared (χ^2^) analyses were used to compare a diagnosis of AHR between lung function testing modalities. The coefficient of variation was calculated for initial baseline measurements (over 3 min) for each horse for the delta flow, theta, and respiratory rate, and the means compared using Mann-Whitney *U*-test. Statistical analyses were performed using statistical software^16^, with *P* < 0.05 considered significant.

## Results

A total of 19 horses met the inclusion criteria and completed the study. Three additional horses were initially enrolled, but excluded due to evidence of either infectious respiratory disease or RAO at the time of examination. These were five mares and 14 geldings, with a mean age of 13 ± 5.5 years, weighing 507 ± 80 kg, in good to obese body condition (median BCS of 6, range 4–9; not recorded in one horse). Exercise level varied, with one racehorse, nine horses in a moderate level of exercise (eventers, showjumpers, etc.) and the remaining nine horses in light work (pleasure riding, etc.). Baseline characteristics are presented in Table 1. All horses were clinically normal at rest.

**Table 1:** Type and duration of clinical signs in horses presented for the evaluation of Equine Asthma (EA).

		**Total**	**EA**	**Non-EA**
		19	14	5
Clinical signs	Poor performance	16 (84%)	11 (79%)	5 (100%)
	Cough	15 (79%)	12 (86%)	3 (60%)
	Nasal discharge	3 (16%)	3 (21%)	0
	Combination	12 (63%)		
Duration of signs	<6 months	10 (53%)	6 (43%)	4 (80%)
	>6 months	9 (47%)	8 (57%)	1 (20%)
	Seasonal	4 (21%)	4 (29%)	0
	Unknown	1	1	0
Respiratory rate (breaths/min)		21	21.6	17.6

*Values are given as number of cases and percentage for clinical signs and duration*.

Equine asthma was diagnosed in 14 horses based on combination of history of exercise intolerance or poor performance along with abnormal BALF cytology, whereas five horses were included in the study with a diagnosis of upper airway dysfunction, obesity (leading to exercise intolerance), exercise induced pulmonary hemorrhage (EIPH), or unknown disease. Of those horses with a diagnosis of equine asthma based on clinical history/examination and BALF cytology, 9/14 (64%) had >5% neutrophils, 9/14 (64%) had >2% mast cells, and 4/14 (21%) had >1% eosinophils. An increase in more than one cell type was seen in 7/14 (50%) study animals, where a high percentage of eosinophils in BALF was always associated with a concurrent elevation in either neutrophils or mast cells.

Baseline respiratory system resistance (FOM) and delta flow (FP) are presented in Table 2. There was no significant difference in resting pulmonary function parameters between horses diagnosed with EA and those with normal BALF cytology. However, FOM testing established a diagnosis of airway hyperresponsiveness (100% increase in R_rs_ from baseline (PC_100_R_rs_) or a clinical reaction at ≤8 mg/mL histamine) in seven horses, all of which had abnormal BALF cytology. Employing a cut point of PC_75_R_rs_ instead of PC_100_R_rs_ did not alter the diagnosis of AHR in any horse. The mean dose of histamine required to reach PC_100_R_rs_ was lower in horses with BALF cytology compatible with equine asthma compared to those with normal BALF cytology but the difference was not significant (Table 2). FP diagnosed AHR based on PC_35_Δ_flow_ ≤6 mg/mL histamine in 11 horses, of which 9/11 (82%) showed an abnormal BALF cytology, and 2/11 (18%) had a normal BALF cytology. As with FOM, the mean histamine dose required to reach PC_35_Δ_flow_ was lower in horses with BALF compatible with EA compared to those with normal BALF cytology, but the difference was not significant (Table 2). Overall, FP was more likely to diagnose AHR in horses with both normal and abnormal BALF compared to FOM (*p* = 0.013). The individual cellular profile of BALF was not statistically associated with a diagnosis of AHR based on FOM or FP (Table 3).

**Table 2:** Pulmonary function test results (median and range) for saline baseline and histamine provocation using FOM and FP.

		**EA**	**Non-EA**	***P*-value**
FOM	Baseline Rrs (cmH_2_0/L/s)	0.46 (0.29–0.79)	0.47 (0.35–0.66)	0.754
	PC_100_R_rs_ (mg/mL)	11.16 (3.51–16)	16 (8-16)	0.107
FP	Delta flow (L/s)	1.02 (0.33–2.94)	1.7 (0.48–3.13)	0.298
	PC_35_Δ_flow_ (mg/mL)	4.78 (0.74–39.85)	13.63 (2.43–36.30)	0.391

*PC, provocative concentration; Rrs, respiratory system resistance*.

**Table 3:** AHR as measured by FOM (PC_100_R_rs_) and FP (PC_35_Δ_flow_) compared to BALF cytology subtypes [median (range)].

**Cytology finding**		**PC_**100**_R_**rs**_ (mg/mL)**	**PC_35_Δ_flow_ (mg/mL)**
Neutrophils	>5%	8 (3.51–16)	5.3 (0.81–39.59)
	<5%	16 (4-16)	5.51 (0.74–36.3)
	*P*-value	0.278	0.604
Mast cells	>2%	15.19 (3.51–16)	5.51 (0.74–30.14)
	<2%	14.32 (4-16)	5.3 (0.86–39.59)
	*P*-value	0.840	0.657

*Eosinophils are not included as too few cases had increased numbers (only two horses)*.

The FP-measured parameter theta (phase angle) was not useful in distinguishing between horses with EA and those without, either during baseline measurements (Table 4) or using histamine provocation. Although theta did increase following histamine administration (saline baseline theta 18.88 ± 8.04; theta after final concentration of histamine 28 ± 14.53; *p* = 0.006), a significant association with percentage increase in theta and EA diagnosis was not present (using theta PC_50_ as a cut off for diagnosis of EA *p* = 0.500). In contrast, initial baseline respiratory rate was significantly different between EA and non-EA horses (*p* = 0.001) with a corresponding decrease in inspiratory and expiratory times in horses with EA (Table 4). Tidal volume was also lower in horses diagnosed with EA (*p* = 0.039). Respiratory rate also increased with histamine challenge as expected but the difference between groups did not reach statistical significance.

**Table 4:** FP derived parameters during initial baseline measurements, showing mean and SD for EA and non-EA horses and the *p*-value comparing the groups.

	**EA mean ± SD**	**Non-EA mean ± SD**	***P-*value**	**Coefficient of variation median (range)**
Delta flow (L/s)	1.19 ± 0.64	1.75 ± 1.04	0.174	0.52 (0.27–38.64)
Theta (degree)	9.85 ± 4.46	11.77 ± 7.10	0.488	0.91 (0.55–4.63)
Respiratory rate (breaths/min)	12.25 ± 3.11	8.48 ± 1.18	0.001	0.11 (0.04–6.99)
Tidal volume (L)	6.17 ± 1.24	7.53 ± 0.94	0.039	0.15 (0.05–1.25)
Minute ventilation (L)	72.63 ± 18.33	63.39 ± 9.33	0.302	0.14 (0.06–11.74)
Peak inspiratory flow (L/s)	4.75 ± 0.79	4.69 ± 0.65	0.888	0.14 (0.06–0.51)
Peak expiratory flow (L/s)	6.38 ± 1.79	7.06 ± 1.80	0.479	0.16 (0.05–2.05)
Inspiratory time (s)	2.22 ± 0.49	3.15 ± 0.47	0.002	0.14 (0.04–0.71)
Expiratory time (s)	3.08 ± 0.77	4.02 ± 0.90	0.038	0.12 (0.06–2.04)

*The coefficient of variation is also included for each parameter*.

Both lung function methods were well-tolerated by all horses. Horses received 0.49 mg/kg (0.33–0.87 mg/kg) xylazine for FOM, and 0.50 mg/kg (0.33–0.99 mg/kg) for FP [median (range)]. Fourteen horses received the same total dose of sedation to perform both testing modalities. The remaining five horses required additional sedation to complete the tests, with one horse requiring a higher total dose of sedation to perform FOM, and the other four horses required a higher total dose of sedation for FP.

## Discussion

This study demonstrates that FOM and FP cannot be used interchangeably to assess AHR in horses as test results are discordant, with AHR diagnosed in 11 horses using FP and in only seven of these horses using FOM. Moreover, FP was more likely to diagnose AHR even in horses without a clinical diagnosis of EA. Although both testing methods have previously been compared to conventional esophageal balloon-pneumotachography (5, 6), the latter testing method was not included in this study of client owned horses due to its degree of invasiveness and lack of use outside of research purposes (18).

The diagnosis of EA in this study was based on a clinical history of poor performance, exercise intolerance, or chronic cough along with abnormal BALF cytology rather than the documentation of AHR. While reliance on clinical history and BALF cytology for the diagnosis of EA is standard practice (1), it has been shown that a subset of horses with clinical signs suggestive of equine asthma but normal BALF cytology, exhibit pulmonary dysfunction based on histamine bronchoprovocation testing (9, 19). Moreover, the documented lack of improvement in BALF-based inflammation in multiple studies suggests that response to treatment should be assessed by PFT and not by re-evaluation of BALF cytology (20–24). These observations support the importance of pulmonary function testing with bronchoprovocation in the management of horses with equine asthma.

Airway hyperresponsiveness was a frequent finding in our study in horses with a complaint of cough or poor performance, with 11/19 [58%] affected, comparable to previous reports [24/45 (53%)] (9). However, a higher percentage of horses showed AHR in the absence of BALF abnormalities in the latter report [11/45 (24%)], compared to our current study [2/19 (11%)]. Various studies have associated increased AHR with increased mast cell concentrations in BALF (14, 25, 26), increased eosinophils (27) or both (28); while others saw no association between cell type and AHR (9, 10). Using open plethysmography in horses without any clinical history or evidence of EA, Cullimore et al. (29) documented AHR in 52% and BALF cytology compatible with EA in 42% of horses. However, AHR and BALF cytology were not correlated, and indeed, 53% of horses with normal BALF cytology demonstrated AHR. The results of our current study do not indicate a relationship between abnormal BALF cytology and AHR, irrespective of the cell type evaluated or testing method used.

The expectation that airway hyperresponsiveness must necessarily be conjoined with airway inflammation has been challenged in humans for some time (30, 31). In fact, recent studies in horses (9, 29) have supported that AHR may not be associated with airway inflammation. Certainly, airway hyperresponsiveness has been observed in non-asthmatic, atopic humans as well as in horses (32). This further underscores the importance of identifying accurate PFT methods for the diagnosis of EA, where both inflammatory and functional disturbances may independently contribute to the disease pathogenesis.

The reason for the difference between the two testing methods (FOM and FP) is not immediately obvious, and the human literature provides ample evidence that varying methods of lung function testing are not necessarily comparable. In people, the forced oscillatory technique was shown to be both more sensitive and more specific than spirometry [forced expiratory volume in one second (FEV1)] in assessing bronchial hyperresponsiveness (33). Likewise, body plethysmography, which is similar but not the same as flowmetric plethysmography (34), demonstrated evidence of AHR in a high number of clinically diagnosed asthmatic individuals who did not reach the diagnostic threshold required by spirometry. A close relationship between the dose-response curves obtained by plethysmography and a forced oscillatory technique was identified in human asthmatics in one study (35). Another study reported higher test sensitivity of body plethysmography compared to impulse oscillometry when investigating both methacholine and allergen challenges in people (36).

It is possible that the definition of AHR within each test requires adjustment of cutpoints (e.g., decreasing the sensitivity of FP by increasing the cutpoint to a 50% increase rather than a 35% increase in delta flow, PC_35_Δ_flow_ vs. PC_50_Δ_flow_), or by similarly increasing the sensitivity of FOM. Looking first at FOM, the results of the current study would not have changed, and the same diagnosis would be made in each case regardless of whether an end point of PC_75_R_rs_ or PC_100_R_rs_ at ≤6 or ≤8 mg/mL was used. With respect to FP, a cut off of PC_35_Δ_flow_ for FP at ≤6 mg/mL histamine was used as this was found to correlate with conventional testing (PC_65_C_dyn_) (6), resulting in 11 horses being diagnosed with AHR. However, had we used a cutpoint of ≤8 mg/mL histamine, three horses with EA based on history/clinical signs and BALF cytology but without AHR at PC_35_Δ_flow_ ≤6 mg/mL histamine, would have been placed in the AHR category. Alternatively, if PC_50_Δ_flow_ had been used (12) at ≤6 mg/mL histamine then one horse with AHR at PC_35_Δ_flow_ ≤6 mg/mL histamine would have been re-classified as normal. Altering the cut point for FP would not have changed the results for the two horses with AHR but normal BALF cytology. Regardless of alternative cutpoints, the lack of correlation between FOM and FP would not have changed.

In the four horses where the diagnosis of AHR differed between FOM and FP, only two were diagnosed with EA based on clinical assessment and BALF cytology. None of the four horses reacted with FOM, all having a PC_100_R_rs_ of >8 mg/mL histamine. Thus, FOM appears to be more specific in the diagnosis of AHR, whereas FP appears to be more sensitive.

Of the two horses not clinically diagnosed with EA, one was extremely over-conditioned (BCS 9/9) and solely presented with a clinical complaint of poor performance. In this case, obesity was determined to be the most likely cause. The second horse may have experienced post-viral AHR without cellular evidence of airway inflammation. The results of pulmonary function testing with FOM vs. PF were discordant in these two horses, with FOM demonstrating a normal baseline respiratory resistance and normal response to histamine (PC_100_R_rs_ = 16 and >8 mg/mL), and FP demonstrating marked hyperresponsiveness of the airways (PC_35_Δ_flow_ = 2.43 and 2.89 mg/mL, respectively). It might be expected that the obese horse would have abnormal pulmonary function, as obesity alters lung and chest properties in humans. The latter is based on both mechanical factors increasing airway closure and gas trapping, and inflammatory factors (such as leptin) increasing airway responsiveness (37–41). This suggests that an obese horse may be more likely to display AHR in the absence of equine asthma, but does not explain the disparity between FP and FOM for the obese horse in this study. Similarly, diverse methods such as body plethysmography and oscillometry yield mixed results in obese children, with an inconsistent finding of increased resistance (42–45). If obesity in this horse led to increased gas trapping, then this would increase the delta flow due to increased gas compression. The principle of plethysmography relies on the intrathoracic organs being incompressible (46), yet an increase in adipose tissues may alter this. An inability of the diaphragm to move caudally due to abdominal fat may also lead to increasing signal from the abdominal contribution to plethysmography flow as the horse attempts to breathe deeper in response to histamine. Altered body shape could also lead to increased likelihood of the bands displacing from their location during calibration. The latter is a significant concern in humans, leading to the sole use of a thoracic band in obese patients (47, 48). Similar to people (47), further evaluation of FP in obese horses is required in order ascertain its reliability.

The second horse without a clinical diagnosis of EA was the oldest horse of the study (21 years). It presented for poor performance and a short duration cough, showing an increased number of lymphocytes on BALF with no other abnormalities. Whilst increased lymphocytes may be an age related change, the degree of increase (72%) is still abnormal (10). The FP test showed a sudden increase in delta flow and theta at 4 mg/mL, but the respiratory rate and tidal volumes were unchanged (6–5 breaths per minute, 8.26–8.47 L respectively). The horse spontaneously returned to normal performance following evaluation with no therapy, and was thought to have suffered from a respiratory virus with prolonged recovery. While exaggerated airway response to non-specific stimuli is well-documented in humans (49), and IAD (mild/moderate equine asthma) has been previously associated with recent exposure to respiratory viruses in horses (12), the reason for the lack of concordance between the two diagnostic tests remains unclear.

The two horses with a clinical diagnosis of equine asthma but with a negative bronchoprovocation test using FOM had only subtle changes on BALF, with one having 6% neutrophils and the other having 5% mast cells. Examining the horse's dose response curve following FP may help explain the test disparity in the first horse (6% neutrophils), which showed a sudden increase in delta flow, theta and respiratory rate after nebulization with 8 mg/mL histamine, at the very start of the 2 min recording period. It is possible that this initial and transient peak was missed when testing with FOM due to the inherently greater time taken to initiate testing immediately following histamine nebulization. This horse, moreover, was one which would have been classified as normal on histamine bronchoprovocation if a cutpoint of PC_50_Δ_flow_ at ≤6 mg/mL of histamine had been used. All other horses with AHR in this study showed a consistent increase in delta flow throughout the recording period. This suggests that an advantage of FP over FOM is the ability to monitor the horse's response to histamine over a more prolonged time period, whereas a short transient peak reaction may be missed when testing with FOM. The final horse (with 5% mast cells) had a gradual increase in delta flow which was consistent throughout each recording period, but showed no change in theta or respiratory rate (respiratory rate 7 breaths/min, tidal volume minimally increased from 4.36 to 5.01 L). A definite reason for the difference in results between testing modalities of AHR could not be identified for this horse.

FOM and FP are testing methods that evaluate different aspects of pulmonary function, which may contribute to the differences in diagnosis of AHR between the two tests. FOM measures changes in oscillatory flow and pressure and, at lower frequencies (i.e., 1–3 Hz), computes respiratory system resistance at the level of the lower airways (18). In contrast, FP computes the difference between plethysmographic and pneumotachograph flows (Δ_flow_), representing gas compression (expiration) and expansion (inspiration), as a measure of airway obstruction (18). FOM, as a non-invasive test of pulmonary function, has been previously validated against more invasive testing techniques (esophageal balloon-pneumotachography), and its use with histamine bronchoprovocation is well-described (5, 13, 25). Although the latter technique is routinely used in our hospital to diagnose EA in horses, FOM testing has specific limitations. Achieving good quality data and a high signal-to-noise ratio at 1 Hz oscillatory frequency (a wavelength that bests reflects lower airway function in large animals) can be challenging, due to interference from the horse's breathing (13). Respiratory interference increases at lower Hz frequencies that approach the horse's spontaneous breathing rate (18). The latter is quantified as coherence based on the measured signal-to-noise ratio, where R_rs_ with a coherence of <0.9 is rejected and eliminated from the analysis (14). This makes the methodology less useful for horses with an abnormal respiratory rate or effort at rest (e.g., RAO or upper respiratory abnormalities), as turbulent airflow and breathing rates approaching the 1 Hz frequency can result in data rejection and incomplete testing. Due to measuring R_rs_ of the entire respiratory system, including upper respiratory tract and the chest wall, abnormalities of the upper airway (such as epiglottic entrapment or laryngeal paresis) may lead to an increased R_rs_ due to increased turbulence in the absence of lower airway disease. Upper airway endoscopy should be used to identify or rule out such conditions. Inclusion of the chest wall in the measurement of R_rs_ is considered a limitation as it decreases the sensitivity (13), however it contributes <10% of the total value and remains constant (18), and as such is likely to have minimal effect on bronchoprovocation. Whilst the requirement for obtaining an appropriate coherence value of ≥0.9 can limit the use of FOM in some patients, it also enables elimination of abnormal and inaccurate breaths (such as sniffing or coughing) from being recorded, thus improving the reliability of the data. FOM is also limited in that it is not portable due to the requirement of pressurized gas, and is a system that is only available in a few centers, as it requires expertise for its use.

Despite its limitations, FOM remains a reliable method of lung function testing in horses that are clinically normal at rest, providing diagnostically useful information in both baseline measurements and following histamine bronchoprovocation. In this study, FOM diagnosed AHR only in horses with abnormal BALF cytology, suggesting reliability of positive results, and providing a clinically useful and non-invasive method of assessing response to treatment in these horses.

Whilst FOM is a useful and reliable testing method, FP has the benefit of portability and ease of testing under field conditions. FP also offers fully automated software requiring minimal training for its use: the latter is also a limitation. Real-time evaluation of breath-by-breath data during testing is not possible, although this is required due to the large number of erroneously recorded breaths [which would be eliminated from analysis using FOM due to poor coherence and from conventional testing by use of “filters” or inclusion criteria (18)]. These breaths often have higher delta flow values and contribute to the software produced PC value. The software also automatically stops the test once PC_35_Δ_flow_ is reached, which could result in erroneously completing the test at an inappropriately low concentration of histamine once final analysis of the data is completed. These concerns could be easily eliminated by alterations in the software. FP is based on the concept of whole body plethysmography, and replacing a box with inductive bands can lead to creation of artifacts. Before commencing testing the bands are calibrated to the pneumotachograph based on inspiratory volume, which may cause an overcorrection resulting in pre-existing abnormalities being less well-defined (6). However, further progression in response to histamine is still detected. This also means that the test may be more reliable for defining lower airway disease in horses with a structural upper airway abnormality, and across horses of different sizes as differences in tidal volume are eliminated, due to the horse acting as its own control. One of the limitations of the bands is movement or slipping following calibration, which is more likely to occur with the abdominal band due to body shape. Caudal movement of this band to a slightly narrower region would result in it being less stretched and therefore having greater movement during breathing and falsely increasing the delta flow. Alternatively, the band could move to an area with increased movement, leading to the same result, or to an area with less movement, falsely decreasing the delta flow. This artifact is reduced by careful placement and monitoring of the bands during testing, with re-calibration if movement of the band is suspected. Whilst the bands are calibrated to the individual horse accounting for differences in tidal volume, and tachypnea alone does not increase delta flow, it has been shown that hyperpnea increases delta flow due to accentuation of intrapulmonary gas compression (6). Progressive hyperpnea could occur during testing, because of mask placement (18), use of xylazine (50), or as a response to histamine nebulization. Thus, the occurrence of hyperpnea during testing could lead to a falsely low PC_35_Δ_flow_ with diagnosis of airway hyperresponsiveness. Monitoring of tidal volume during testing would thus be useful. Whilst FP has its limitations when used as a fully automated system, it provides useful information if the data is critically evaluated. Experience with its use eliminates some of the inaccuracies and artifacts that can occur.

One of the significant benefits of FP is the information provided alongside delta flow. The slightly increased minute ventilation, respiratory rate, and peak inspiratory flow, alongside a slight decrease in expiratory time observed in horses with mild/moderate equine asthma in this study is consistent with findings in horses with severe equine asthma (RAO) (15). Although the subtle differences between horses with equine asthma and normal horses may not allow for diagnosis based on this alone, it provides valuable additional information for both clinical evaluation and research.

It has been suggested that theta (phase angle) can be used to measure more subtle changes in thoracic and abdominal contributions to breathing pattern for the assessment of AHR. However, a previous study in foals (16) found no correlation between phase angle (theta) and other measures of lower airway constriction. In contrast, phase angle was significantly increased in horses with heaves (15) compared to normal controls. The latter study also identified that histamine bronchoprovocation led to variable alterations in phase angle in normal horses. However, in the present study the high variability within patients during baseline recording and following histamine administration, suggests that these values may not be accurate using the algorithm currently employed by the software. This is likely because the more the respiratory pattern deviates from sinusoidal, the less accurate Lissajous figure analysis is in estimating phase difference in thoracoabdominal synchrony. Examination of the respiratory pattern in all horses in this study showed a significant deviation from sinusoidal motion which would therefore render inaccurate the embedded Lissajous figure analysis (51). It is therefore not surprising that results of our current data and previous reports suggest that phase angle (theta) cannot be used to reliably assess AHR in response to histamine bronchoprovocation in horses with clinical signs suggestive of EA.

Limitations of this study include small sample size, potential confounding due to sedation, and potential variability of histamine dose. Sedation with xylazine, required for both forms of PFT used in this study, causes bronchodilation (52) and does affect lung mechanics in horses. Xylazine, however, does not block the bronchoconstrictive effects of histamine. This effect was mitigated as much as possible by standardizing with the same starting dose for each testing method (14). Also, following sedation each horse's head position was maintained in a neutral position (53), which may actually be easier to consistently achieve in the sedated horse (14). Using FOM, R_rs_ at 1 Hz frequency is stable for up to 45 min following xylazine administration at a similar dose used in this study (54). Thus, the effect on testing with FOM was likely minimal. However, other changes in respiratory parameters such as a decrease in respiratory rate and minute ventilation with an increase in tidal volume (50) may have had a greater effect on FP. Whilst a comparison of FOM in sedated and non-sedated horses has been performed (55), all studies using FP have used sedation in order to perform testing. The direct effect of xylazine on delta flow has therefore not been evaluated.

Histamine was nebulized over 2 min, as previous described for both FOM and FP (4–7, 9, 10, 14–16, 26). However, this may lead to variability of dose for individuals, or to different histamine doses for the same individual on consecutive days, since variations of tidal volume or respiratory rate may change the total amount of histamine inhaled (7). Although this is a limitation, it is likely that each horse responded similarly to nebulization on consecutive days, thus comparison of testing in each horse is still reliable.

## Conclusion

Our study indicates that histamine bronchoprovocation using FP establishes a diagnosis of AHR more frequently than does testing with FOM, but that FOM is more likely to accord with a cytological diagnosis of equine asthma. As such, the two pulmonary function testing methods cannot be used interchangeably without further adjustments in current test algorithms or assessment of the effect of xylazine and hyperpnea on delta flow. Further evaluation of the use of FP in specific subgroups (such as obesity) may also be indicated.

## Data Availability Statement

The datasets generated for this study are available on request to the corresponding author.

## Ethics Statement

The animal study was reviewed and approved by Clinical Sciences Research Committee (CSRC) at Cummings School of Veterinary Medicine. Written informed consent was obtained from the owners for the participation of their animals in this study.

## Author Contributions

CD, DB, and MM were involved in the experimental design, patient recruitment, data collection, analyses, and manuscript writing. All authors contributed to the article and approved the submitted version.

## Conflict of Interest

The authors declare that the research was conducted in the absence of any commercial or financial relationships that could be construed as a potential conflict of interest.

## Footnotes

^1^Xylazine 100 mg/mL, AnaSed LA, VetOne, Boise, ID.

^2^Phenylephrine HCl 1%, Nose Drops, Cardinal Health, Dublin, OH.

^3^Fleisch, No 4, OEM Medical, Lenoir, NC.

^4^DP45-28, Validyne Engineering, Northridge, CA.

^5^Open Pleth 4.6.7.0, Ambulatory Monitoring Inc.

^6^ProNeb Turbo, Model 38B0201, Pari, Midlothian, VA.

^7^LC Plus, Pari.

^8^Histamine bisphosphate monohydrate, Sigma-Aldrich, St. Louis, MO.

^9^Detomidine 10 mg/mL, Dormosedan, Zoetis Inc., Kalamazoo, MI.

^10^Butorphanol 10 mg/mL, Torbugesic, Zoetis Inc., Kalamazoo, MI.

^11^Bivona Medical Technologies, Gary, IL.

^12^3 m length 12.9 outer diameter Endoscope, Fujinon, Wayne, NJ.

^13^Lidocaine HCl 2%, Hospira Inc., Lake Forest, IL.

^14^Normal saline solution, VEDCO Inc., St. Joseph, MO.

^15^EasyVac, Precision Medical Inc., Northampton, PA.

^16^SAS, SPSS.
